# Supplementary material for: Functional Analysis of Malus halliana WRKY69 Transcription Factor (TF) Under Iron (Fe) Deficiency Stress
Source: Curr Issues Mol Biol. 2025 Jul 21;47(7):576. doi: 10.3390/cimb47070576 (PMC12293516; doi:10.3390/cimb47070576)
Supplement: Supplementary file 1 [file cimb-47-00576-s001.zip › cimb-3620475-supplementary.pdf]

current issues in molecular biology

Supplementary Table S1. Primers used in the experiment.

Primer	Sequence (5′-3′)
WRKY69-like-F (RT-PCR)	ATGGAGGACTCACCCTCCTCA
WRKY69-like-R	TTATCCTGTATTACTACAATAGGGAACCA
PRI101-WRKY69-like-F	CATATGCCCGTCGACCCCGGGATGGAGGACTCACCCTCCTCA
PRI101-WRKY69-like-R	TCAGAATTCGGATCCGGTACCTTATCCTGTATTACTACAATAGGGAACCA
WRKY69-like-F (qRT-PCR)	TCACCGCTACCGCTACATCAC
WRKY69-like-R	AGTCGTCGCTGAGATCAAGGTC
GAPDH-F	TGAGGGCAAGGTGAAGGGTATCTT
GAPDH-R	TCAAGTCAACCACACGCGTACTGT
